# Age Effects on Women’s and Men’s Dyadic and Solitary Sexual Desire

**DOI:** 10.1007/s10508-022-02375-8

**Published:** 2022-08-02

**Authors:** Larissa L. Wieczorek, Meredith Chivers, Monica A. Koehn, Lisa M. DeBruine, Benedict C. Jones

**Affiliations:** 1 Institute of Psychology, Educational Psychology and Personality Development, University of Hamburg, Von-Melle-Park 5, 20146 Hamburg, Germany; 2 Department of Psychology, Sexuality and Gender Lab, Queen’s University, Kingston, Canada; 3 Discipline of Psychology, Faculty of Health, University of Canberra, Canberra, Australia; 4 School of Psychology & Neuroscience, University of Glasgow, Glasgow, Scotland; 5 School of Psychological Sciences and Health, University of Strathclyde, Glasgow, Scotland

**Keywords:** Sexual desire, Solitary desire, Dyadic desire, Age, Gender/sex effects

## Abstract

While most studies on sexuality in later life report that sexual desire declines with age, little is known about the exact nature of age effects on sexual desire. Using self-reported dyadic sexual desire relating to a partner, dyadic sexual desire relating to an attractive person, and solitary sexual desire from a large (*N* > 8000) and age diverse (14.6–80.2 years) online sample, the current study had three goals: First, we investigated relationships between men and women’s sexual desire and age. Second, we examined whether individual differences such as gender/sex, sexual orientation, self-rated masculinity, relationship status, self-rated attractiveness, and self-rated health predict sexual desire. Third, we examined how these associations differed across sexual desire facets. On average, the associations between age and both men and women’s sexual desire followed nonlinear trends and differed between genders/sexes and types of sexual desire. Average levels of all types of sexual desire were generally higher in men. Dyadic sexual desire related positively to self-rated masculinity and having a romantic partner and solitary desire was higher in people with same-sex attraction. We discuss the results in the context of the evolutionary hypothesis that predict an increase of sexual desire and female reproductive effort prior to declining fertility. Our findings both support and challenge beliefs about gender/sex specificity of age effects on sexual desire and highlight the importance of differentiating between desire types.

## Age Effects on Women’s and Men’s Dyadic and Solitary Sexual Desire

Sexual desire can be understood as the experience of sexual thoughts, fantasies, and the motivation to engage in sexual activity (Basson, 2002). Given the positive link between sexual desire and general well-being (e.g., Davison et al., 2009; Robinson & Molzahn, 2007; Willert & Semans, 2000), it is important to understand the factors that predict sexual desire across the life course. Several studies suggest that, on average, sexual desire is negatively associated with age (e.g., Dawson & Chivers, 2014; DeLamater & Sill, 2005; Laumann et al., 2005; Lindau et al., 2007). Looking at gender-/sex-specific effects, Alfred Kinsey proposed that “the male may be most desirous of sexual contact in his early years, while […] most females become less inhibited [over the years] and develop an interest in sexual relations, which they may maintain until they are in their fifties or sixties” (Kinsey et al, 1953, pp. 353). This prediction has seldom been questioned (see Barr et al., 2002), yet assumptions regarding the specific age when women’s desire is highest are more diverse. While no certain point in life is known to mark a change in sexual functioning for men, the transition into menopause is associated with a decrease in sexual functioning in women (Dennerstein et al., 2001; Petersen & Hyde, 2011). Given these age-related biological changes, evolutionary psychologists have hypothesized that women would experience an increase of sexual desire around age 35 to maximize their reproductive output before they lose their fertility (Easton et al., 2010; Schmitt et al., 2002). In Barr et al.’s (2002) survey on socially shared cognitions regarding women and men’s sexual peak, participants expected female desire to be highest around age 27 and male desire to be highest around age 22. Finally, media, such as the popular TV series *Sex and the City,* paint a picture of women who have adventurous sexual lives and whose desire remains high through and after their thirties.

Despite these common assumptions on gender-/sex-specific age effects on sexual desire—that women reach their peak of sexual desire later than men do—corresponding scientific evidence is lacking. Importantly, human sexuality is widely conceptualized as a product of fundamentally entwined biological and sociocultural influences (Fausto-Sterling, 2005; Levine, 2003). Thus, a wide range of biological and psychosocial factors should be considered when studying age effects on sexual desire. The current study had three objectives: First, we examined sexual desire across women and men covering a broad age range to empirically evaluate gender-/sex-specific age effects on sexual desire. Second, we investigated which biological and psychosocial factors (i.e., gender/sex, sexual orientation, self-rated masculinity, relationship status, self-rated health and self-rated attractiveness) relate to sexual desire. Third, we examined how the associations between sexual desire and the remaining study variables differed across specific facets (Moyano et al.,; 2017; Spector et al., 1996) of sexual desire.

## Facets of Sexual Desire

The Sexual Desire Inventory-2 (SDI-2; Spector et al., 1996) is the most commonly used instrument for the measurement of trait sexual desire in normative circumstances (see Dawson & Chivers, 2014; Stark et al., 2015; Toledano & Pfaus, 2006). In its original conceptualization (Spector et al., 1996), the SDI-2 consists of two facets measuring *dyadic sexual desire* (i.e., desire for partnered sexual activity) and *solitary sexual desire* (i.e., desire for solitary sexual activity, like masturbation). As noted by Holmberg and Blair (2009), however, it might be more reasonable to split the dyadic sexual desire scale into items referring to a (current) sexual “partner” or to an “attractive person,” suggesting desire toward an acquaintance or stranger. Conceptually, these two facets of dyadic desire differ from each other in that desire relating to a partner strongly depends on characteristics of the romantic partner or relationship, whereas desire relating to an attractive person may occur independent of a person’s current relationships (Moyano et al., 2017). Moreover, the two facets of dyadic sexual desire and corresponding sexual behaviors reflect different psychological needs, which likely vary across the life span. Specifically, having sex with a romantic partner might follow the wish to express love and to feel connected, whereas having sex with an attractive person might follow the wish to experience sexual variety (Meston & Buss, 2007).

Using exploratory and confirmatory factor analyses in two samples of heterosexual men and women in committed romantic relationships (*N*_overall_ = 4,094), Moyano et al. (2017) provided empirical support for the distinction of dyadic sexual desire into *dyadic desire (partner)* and *dyadic desire (attractive person)*. These further specified scales have also been validated in a Columbian sample (Vallejo-Medina et al., 2020) and a sexual minority sample (Mark et al., 2018). Moreover, Moyano et al. (2017) found that whereas dyadic desire (partner) related to higher sexual satisfaction, dyadic desire (attractive person) related to higher tendency to become sexually aroused. This way, both theoretical and empirical research suggests a differential relevance of dyadic desire (partner) and dyadic desire (attractive person). Given that our original hypotheses were based on the distinction between the broader constructs of dyadic and solitary sexual desire, however, differences between these two more specific sexual desire facets will be examined in an exploratory manner.

## Factors Influencing Sexual Desire

### Age

Age is typically negatively related to sexual desire (Beutel et al., 2008; DeLamater & Sill, 2005; Eplov et al., 2007). Apart from this, less is known about variation in sexual desire from early to late adulthood or how these age effects might vary by gender/sex. Of note, age effects on sexual desire need to be understood as a complex interplay of biological and psychosocial factors (Levine, 2003; Tolman & Diamond, 2001). For example, the wish to find a partner or to become a parent can increase an individuals’ sexual desire in young years (Levine, 2003). In contrast, sexual desire might be diminished at higher ages by the loss of one’s romantic partner (Kontula & Haavio-Mannila, 2009) or stigmatization of sex among the elderly (DeLamater & Sill, 2005). Despite the undeniable relevance of these psychosocial aspects, gender-/sex-specific predictions are mainly based on biological explanations.

Menopause, defined as cessation of menstruation for one year, usually occurs between ages 45 and 55 (National Health Service, 2017), yet women’s fertility is believed to start declining at age 32, and more rapidly after age 37 (Practice Committee of the American Society for Reproductive Medicine, 2008). The age of 35 therefore represents a marker of a life phase where female reproductive capacity decreases, but fertility persists for approximately another decade. Men’s fertility, in contrast, is only barely affected by age (Dunson et al., 2002). Given these gender/sex specificities in age-related changes of fertility, evolutionary psychologists theorized that women’s sexual desire should increase in the years prior to declining fertility to maximize the probability of reproduction (Buss, 2016). In contrast, no such distinct patterns are predicted for men. In line with this hypothesis, Schmitt et al. (2002) found that women aged 30–34, compared to younger (aged 18–24) and older (aged 35–54) women, showed higher levels of desire. For men, Schmitt et al.’s results indicated a peak of sexual desire that occurs at age 25–29, and thus earlier than for women. In a study by Easton et al. (2010), women aged 27–45 reported higher desire than younger (aged 18–26) and older (aged 46 and older) women; no men were included in this study. Whereas these studies provide first empirical support for the hypothesis that women’s sexual desire is highest before fertility declines and men’s desire is highest at a younger age, it is important to note some limitations: First, given that only few participants aged 30–34 (*N*s ranging from 14 to 53) were included in Schmitt et al.’s (2002) study, the comparison between this group and remaining participants was likely underpowered. Second, in Easton et al.’s (2010) study, the lack of male participants makes gender/sex comparisons impossible. Third, in both studies, the use of data aggregated in age groups complicates a more nuanced interpretation on how sexual desire relates to age. Thus, findings need to be replicated with a larger sample involving both women and men and using a nuanced measure of age.

Overall, extant research suggests a negative relationship between sexual desire and age, but that specific effects might differ by gender/sex. Accordingly, we expected that, on average, sexual desire is negatively associated with age (Hypothesis 1a). Moreover, we predicted that women’s sexual desire is highest between ages 35 to 45, but negatively associated with age thereafter (Hypothesis 1b), while men’s sexual desire is negatively associated with age in a linear way (Hypothesis 1c).

### Gender/Sex

Previous studies typically report lower sexual desire among women (e.g., Kim et al., 2021; Lippa, 2009; Sutherland et al., 2015), especially when solitary desire (e.g., masturbation) is measured (Baumeister et al., 2001; Hyde, 2005; Stark et al., 2015). Several theories have been proposed to explain the gender/sex difference in trait sexual desire. Among the most influential are sexual strategies theory (Buss & Schmitt, 1993), and social theories, including social learning theory (Bandura, 1986). Sexual strategies theory proposes that males should, on average, be more interested in a higher number of short-term mates, while females should, on average, be more interested in acquiring reliable long-term mates due to their relatively higher reproductive costs (Buss & Schmitt, 1993; Gangestad & Simpson, 2000). Higher sexual desire would therefore function as an evolutionary adaptation that helps men to increase their reproduction rate, while the lower desire would help women to only invest in offspring with suitable partners. According to social learning theory, gender/sex differences in (expressions of) sexual desire follow social learning from different behavior of same gender/sex role models in real life and media (Bandura, 1986; Chivers, 2014). In addition, less reinforcement or more punishment for women expressing sexual desire is believed to amplify gender/sex differences (Bussey & Bandura, 1999; Petersen & Hyde, 2011).

Together, both theory and previous studies suggest higher sexual desire in men. At the same time, such gender/sex effects might vary across sexual desire facets and various biological and social variables are likely to contribute to this difference. Thus, we expected that, on average, men report higher sexual desire than women (Hypothesis 2a), while men and women’s dyadic desire is more similar than their solitary desire (Hypothesis 2b).

### Sexual Orientation

Sexual orientation effects on sexual desire have also been suggested, yet empirical evidence is mixed. On the one hand, data by Lippa (Lippa, 2006, 2007) indicated that gay and bisexual men had somewhat lower sex drive than heterosexual men, whereas bisexual women were higher in sex drive than heterosexual and lesbian women were. On the other hand, recent findings suggest that gay men score higher on solitary and dyadic (attractive person) sexual desire (Peixoto, 2019). Thus, whereas sexual orientation effects on sexual desire remain a topic for investigation, other findings from the sexuality research might inform about corresponding associations: While it is more socially accepted for men than for women to express sexual desire in general (Petersen & Hyde, 2011), women with same-sex attraction are more likely to deviate from heteronormative mating scripts that envisage a rather passive role for women (Jackson, 2006). These women might have learned to express their sexual desire to a greater degree than typically reported by heterosexual women. Also, findings from studies examining sexual concordance (i.e., the agreement between genital and self-reported sexual arousal; Suschinsky et al., 2017) point to the possibility that same-sex attracted women have learned to register their sexual arousal more precisely than heterosexual women and therefore experience more sexual desire (Everaerd & Both, 2001; Meana, 2010).

Overall, it seems likely that sexual orientation differences in sexual concordance and socialization experiences are reflected in different levels of sexual desire, especially among women. Integrating these different results and assumptions, we expected that same-sex attracted and heterosexual men show higher sexual desire than same-sex attracted women, who show higher sexual desire than heterosexual women do (Hypothesis 3).

### Masculinity

To our knowledge, no study has examined how self-rated masculinity and sexual desire are related. Given that self-rated masculinity is related to sexual identity (Garcia & Carrigan, 1998), gender/sex and sexual orientation (Lippa, 2008), and social scripts and expectations (Eagly & Wood, 1999), we investigated its role in predicting sexual desire. The expression of sexual desire is usually considered male-typical or masculine within a double standard (Petersen & Hyde, 2011; Tolman & Diamond, 2001). Importantly, masculinity varies not only between but also within genders/sexes and many gender/sex differences might arise from femininity and masculinity (Vanwesenbeeck, 2009). In that sense, more masculine men and women could be expected to express higher levels of sexual desire. Moreover, a higher valuation of masculine characteristics in (Western) society potentially facilitates a transgression into masculinity by women and makes a transgression into femininity by men more difficult (Sandford, 2005). These initial findings suggest a masculine connotation of sexual desire and that women might vary more so on the femininity–masculinity continuum than men. Therefore, we expected that self-rated masculinity does positively relate to sexual desire (Hypothesis 4a) and that this effect is stronger for women (Hypothesis 4b).

### Relationship Status

In previous studies, women’s sexual desire was higher in the context of a relationship, whereas male desire was unaffected by these circumstances (Impett et al., 2014; Petersen & Hyde, 2011). At the same time, enduring long-term relationships dampen the sexual desire of both genders/sexes, but especially so for women (Dawson & Chivers, 2014; Klusmann, 2002; McNulty et al., 2019; Meana, 2010; Murray & Milhausen, 2012). Using two large national samples in Finland (*N*_overall_ = 3,682), Kontula and Haavio-Mannila (2009) found that relationship duration had no effect on men’s and women’s sexual desire when controlling for other factors, such as sexual functioning. Looking at later life (age 65 or older), having a sexual partner is a strong predictor for having sex and sexual desire (DeLamater & Sill, 2005; Kontula & Haavio-Mannila, 2009), while widowed women (who are more common than widowed men because more women partner with older men) might have lower (dyadic) desire as an adaption to the lack of access to sexual partners. In turn, having a partner likely reduces solitary sexual desire, since one reason for feeling the desire to masturbate can stem from the unavailability of a partner (Carvalheira & Leal, 2013; Reece et al., 2010). Given these observations, we examined the effects of relationship status on the types of sexual desire, and their interaction with age. Whereas previous evidence for relationship status effects on sexual desire is mixed, associations might differ across sexual desire facets and be moderated by age. Accordingly, we predicted that having a partner relates positively to dyadic sexual desire and negatively to solitary sexual desire (Hypothesis 5a) and that the interaction between relationship status and age positively predicts dyadic sexual desire (Hypothesis 5b).

### Health and Attractiveness

Unsurprisingly, clinicians consider health among one of the key variables shaping sexual desire (Levine, 2003) and numerous studies provide empirical support for this link (e.g., Dennerstein et al., 1999; Laumann et al., 1999; Shifren et al., 2008). Age-related decreases of sexual desire can partly be attributed to poorer health, including physiological changes that alter sexual functioning (Willert & Semans, 2000; Kontula & Haavio-Mannila, 2009). Next to health, self-rated attractiveness might be an important predictor of sexual desire as related variables such as poor body image and low sexual self-esteem are frequently listed as factors contributing to sexual functioning concerns, including low sexual desire in women (e.g., Koch et al., 2005; Kontula & Haavio-Mannila, 2009; Seal et al., 2009). Moreover, objectification theory (Fredrickson & Roberts, 1997) suggests higher levels of self-consciousness and critical self-observation among women. Therefore, self-rated attractiveness might have a greater impact on female than male desire. Given these findings, we examined the effects of self-rated health, self-rated attractiveness and of the interaction between gender/sex and self-rated attractiveness on sexual desire in the current study.^1^

## Current Study

The current study aimed to examine relationships among age and sexual desire using over 8000 participants’ responses to the Sexual Desire Inventory-2 (Spector et al., 1996) in an online survey. The impact of various additional biological and psychosocial factors, including gender/sex, sexual orientation, self-rated masculinity, relationship status, self-rated health, and self-rated attractiveness, on sexual desire were also examined. Hypotheses and analyses were preregistered at https://osf.io/f7hsn via the Open Science Framework (Center for Open Science, 2011–2022). Whereas some of our preregistered hypotheses refer to *dyadic* sexual desire, findings by Moyano et al. (2017) and results of confirmatory factor analyses with our own data (see Appendix A), suggested that a differentiation between *dyadic desire (partner)* and *dyadic desire (attractive person)* in addition to *solitary desire* would be more appropriate. Accordingly, in this article, we report separate effects for both dyadic sexual desire facets. Nonetheless, results from the analyses that were based on the original SDI-2 facets (Spector et al., 1996) can be retrieved from Appendix B. For reasons of parsimony, only the most relevant hypotheses and corresponding results are presented in the main part of the article, but results from all preregistered analyses (i.e., analyses on self-rated attractiveness, mediation effects that might account for gender/sex differences, and effects of hormonal contraception) are reported in Appendix C.

## Method

### Participants

Data collection was part of a larger online study on social attitudes and personality conducted by [names masked for review], which was administered via the Experimentum platform (DeBruine et al., 2020). The study was advertised via a number of social media platforms (e.g., stumbleupon.com). Participants took part on a voluntary basis and were recruited between July 2007 and March 2018.^2^ In the original study, *N* = 8205 participants provided data about their sexual desire and were therefore considered to be included in the current study. From this original sample, participants providing no information about their gender/sex (*n* = 42) or age (*n* = 5) were excluded. Further, participants with an unrealistically high age (> 100; *n* = 5) and intersex or non-binary individuals (*n* = 3) were excluded due to their small number and the relevance of gender/sex in all research questions. After applying the exclusion criteria, the remaining total sample consisted of *N* = 8150 participants that were aged between 14.6 and 80.2 years (*M* = 24.89, *SD* = 7.91) and consisted of a larger portion (67.88%) of women. As indicated by the mean age, most participants were relatively young in comparison to the large age range. With regard to sexual orientation, 5882 (72.17%) identified as heterosexual, 1600 (19.63%) indicated same gender/sex attraction (i.e., attraction to the same gender/sex or to both men and women), 25 (0.31%) indicated asexual orientation and the rest indicated no sexual preference. At the time of measurement, 2416 (29.64%) of the participants had a partner, 2295 (28.16%) were single, and the remaining participants did not provide any information about their relationship status.

**Fig. 1:**
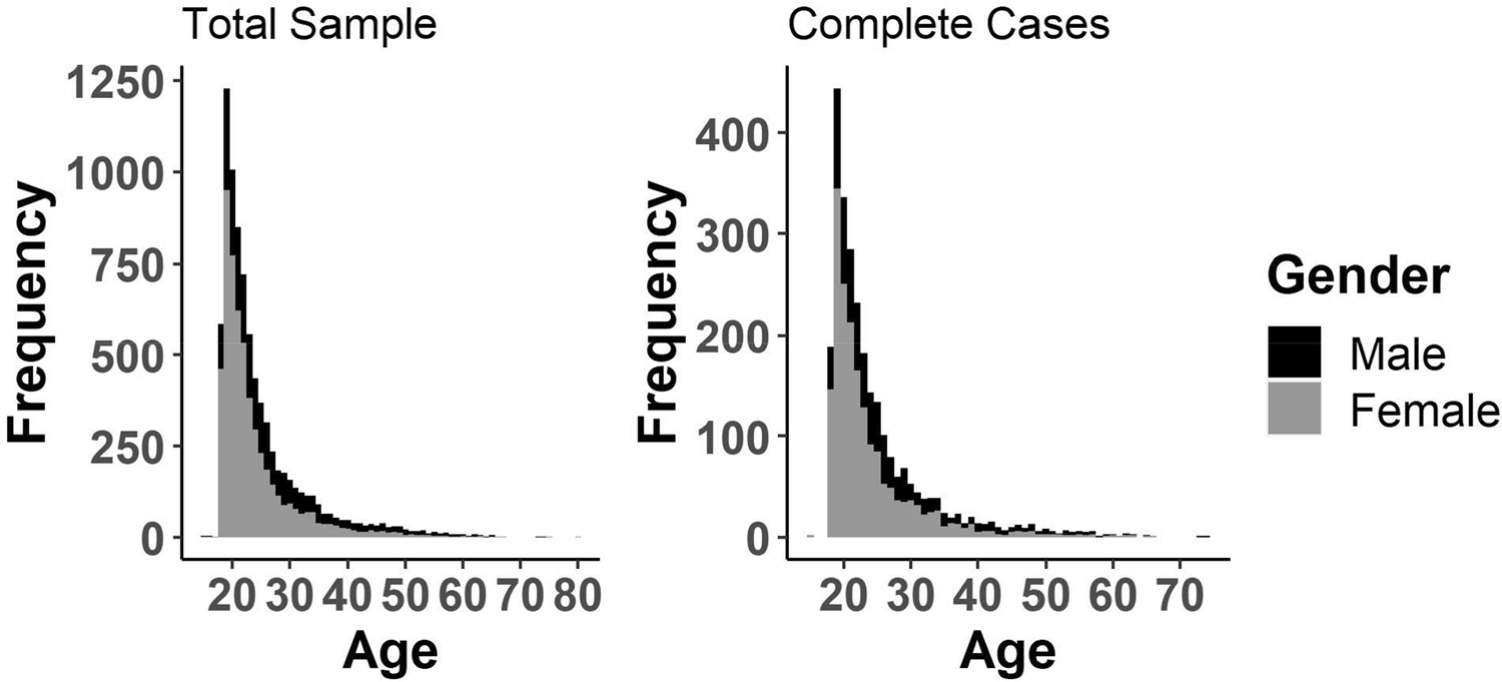
Age Distribution. *N*_Total Sample_ = 8150, *N*_Complete Cases_ = 2744

Since using an existing data set, the sample size was predetermined. Nonetheless, we performed sensitivity analyses with the software G*Power (Faul et al., 2007) to determine whether our sample had sufficient power (95% power at an alpha error-level of α = 0.05) to detect small effects (*f*^*2*^ = 0.02 or *d* = 0.20). A subsample of *N* = 2744 participants provided complete answers on all study variables. As for the total sample, the expected sensitivity (95% power to detect small effects) in our analyses remained high. Compared to the total sample, participants providing complete information on all variables was more likely to be non-heterosexual, *t*(4735.3) = 2.37, *p* = 0.018, *d* = 0.05, 95%CI [0.01, 0.10], and less likely to rate themselves as healthy, *t*(5767.7) = –2.03, *p* = 0.042, *d* = –0.05, 95%CI [–0.10, –0.00], with negligible effect sizes. With regard to age, gender/sex, relationship status, self-rated attractiveness, self-rated masculinity, and all sexual desire facets, no difference was found. The age distribution of our total sample and the subsample of complete cases is illustrated in Fig. 1.

### Procedure and Measures

The [institution masked for review] has approved data collection. All measures were assessed via self-reports during an online survey with the order of questionnaires randomized across participants. The measures that were identified as being relevant to research questions concerning sexual desire over the life course und used in this study are described in the following:

#### Demographic and Identity Measures

Participants indicated their gender/sex with one of the answer options (“male,” “female,” “prefer not to answer” or “does not apply to me”), their age, and their relationship status as being in a relationship or not. In addition, sexual orientation was captured by indication of a sexual preference for men, women, any (e.g., bisexual), or none (e.g., asexual). For all participants except those who answered “none,” a new variable coding their sexual orientation as heterosexual (attracted to the opposite sex) or same gender/sex attracted (attracted to the same gender/sex and both gender/sexes) was computed.

#### Sexual Desire

Sexual desire was measured with the SDI-2 (Spector et al., 1996; see Table 6 for items). Dyadic sexual desire (partner) was measured with seven items, dyadic sexual desire (attractive person) with two items, solitary sexual desire with four items, and the total score of sexual desire with all fourteen items. Answers were given on 9-point scales ranging from 1 to 9 in the case of items assessing strength of sexual desire and on 8-point scales ranging from 0 to 7 in the case of items assessing frequency of sexual desire. The anchor labels of the SDI-2 scales varied by item, but a higher value reflected higher/ more frequent sexual desire in every item. Internal consistencies as indicated by Cronbach’s α in our study were 0.88, 0.84, and 0.92 for dyadic (partner), dyadic (attractive person), and solitary sexual desire, respectively.

#### Self-Ratings

Participants rated their own masculinity, attractiveness, and health on a 7-point scale ranging from 1 (*much less masculine/attractive/ healthy than average*) to 7 (*much more healthy/attractive/masculine than average*). Similar measures have been successfully used in research on vocal and facial partner preferences (e.g., Feinberg et al., 2012; Kandrik & DeBruine, 2012).

### Data Analysis

All analyses were run with the statistical open source program R version 3.6.0 (R Core Team, 2021), RStudio (RStudio Team, 2021) and several R packages (Revelle, 2018; Tingley et al., 2014; Torchiano, 2016; Wickham, 2011; Wickham et al., 2018). Figures were generated using the R package ggplot2 (Wickham, 2016). When possible, the whole sample (*N* = 8150) was analyzed as all participants completed the SDI-2 and provided information on gender/sex and age (see exclusion criteria above for additional information). If participants provided incomplete information, they were excluded from affected hypotheses testing but included in the remaining, unaffected analyses. Therefore, used sample sizes varied depending on the variables included in the analyses. In addition, participants that answered “none” (e.g., this could translate to “asexual”) in response to the question about their sexual preference were excluded from analyses involving sexual orientation, because they were too few (*n* = 25).

Since all reported hypotheses included sexual desire as the dependent variable, and in order to control for the effects of the other predictor variables, many of the hypotheses were tested with the same large multiple regression models (*full models*) based on the subsample that provided complete answers on all variables included (*N* = 2744). In the full models, total and subscale SDI-2 scores were predicted from age, gender, sexual orientation, self-rated masculinity, self-rated health, self-rated attractiveness, relationship status, and the interactions between age and relationship status, gender/sex and self-rated attractiveness, gender/sex and self-rated masculinity, and gender/sex and sexual orientation. In addition to analyzing predictor effects in the full models, the hypothesized relationships between age, gender/sex and sexual orientation, and self-rated masculinity with sexual desire were further examined in linear and polynomial trend analyses, t-tests, and mediation analyses, respectively. All metric measures in the regression models were *z*-standardized. The code and the data that are necessary to reproduce all results can be retrieved from https://osf.io/rba2x.

We deviated from our original analysis plan in three aspects. First, some errors in our analysis script led to wrong computations of (sub)sample sizes for power estimation: In the preregistration, the total sample size was stated as *N* = 8146 instead of the actual *N* = 8150 and the size of the subsample with complete information on all measures was stated as *N* = 3476 instead of the actual *N* = 2744. Second, given our large sample size and the relatively high number of analyses, we have subsequently decided to interpret effects with *p* values < 0.001 instead of < 0.05, as originally stated, in order to account for the heightened risk of false positive findings (e.g., Kaplan et al., 2014). Finally, we used bootstrapping method to test whether effect sizes obtained in t-tests significantly differed from each other instead of simply comparing confidence intervals.

## Results

Descriptive statistics and bivariate intercorrelations among continuous study variables can be found in Table 1 for men and women separately. For both men and women, all types of sexual desire were positively related to each other, with higher correlations between the two facets measuring dyadic sexual desire.

**Table 1:** Descriptive statistics and intercorrelations of the continuous study variables by gender/sex

	*M*	*SD*	Intercorrelations							*N*
			1	2	3	5	6	7	8	
Men										
1. Age	27.34	9.48								2618
2. SDI: Total	71.75	17.30	**.12**							2618
3. SDI: Dyadic-P	38.39	9.78	**.09**	**.85**						2618
5. SDI: Dyadic-A	10.50	3.71	**.08**	**.69**	**.53**					2618
6. SDI: Solitary	18.38	7.41	**.10**	**.72**	**.30**	**.34**				2618
7. Attractiveness^a^	4.62	1.29	− .02	**.10**	**.14**	**.09**	− .01			1525
8. Masculinity^a^	4.12	1.34	**.13**	**.14**	**.22**	**.14**	− .04	**.31**		1525
9. Health^a^	4.65	1.41	.02	.02	.08	.06	** − .10**	**.42**	**.33**	1239
Women										
1. Age	23.74	6.75								5532
2. SDI: Total	61.89	19.90	.03							5532
3. SDI: Dyadic-P	35.76	11.07	− .00	**.86**						5532
5. SDI: Dyadic-A	8.05	4.02	− .03	**.62**	**.45**					5532
6. SDI: Solitary	14.76	8.80	**.06**	**.76**	**.38**	.**33**				5532
7. Attractiveness^a^	4.48	1.29	.04	**.17**	**.17**	**.12**	**.08**			3530
8. Masculinity^a^	3.08	1.53	.02	.05	− .01	.04	**.09**	** − .06**		3510
9. Health^a^	4.17	1.34	.02	.01	**.06**	.03	** − .06**	**.36**	− .04	3168

SDI-2 = Sexual Desire Inventory-2 (Spector et al., 1996); dyadic-P = dyadic (partner); dyadic-A = dyadic (attractive person). All measures were assessed via self-reports. Correlations in bold font are significant (*p* < .001, two-tailed). ^a^Self-rated

### Full Models

All predictor variables were entered into the full models, together with the interactions between age and relationship status, gender/sex and self-rated attractiveness, gender/sex and self-rated masculinity, and gender/sex and sexual orientation (for an overview on the single associations between each variable and total sexual desire, results from simple regression models can be obtained from Table 18*)*. Four full models were run with the data of a subsample of participants that provided answers on all included measures, predicting the total, dyadic (partner), dyadic (attractive person) and solitary SDI-2 scores. The results of the full models are summarized in Table 2. Since the total desire score is an aggregate of the dyadic and solitary desire scores (Spector et al., 1996) and its information is less specific for that reason, we focus on dyadic (partner and attractive person) and solitary sexual desire only. Nonetheless, we report results concerning all four scores.

**Table 2:** Coefficients of the multiple regression models (full models) predicting the SDI-2 scores

	SDI-2 score			
Predictor	Total	Dyadic-P	Dyadic-A	Solitary
Age	.10**	.11***	− .00	.09**
Gender/sex	–.31***	–.07	− .46***	− .28***
Sexual orientation	.31***	.15	.13	.42***
Masculinity ^a^	.18***	.21***	.17***	.06
Relationship status	.13***	.33***	− .16***	− .11**
Health ^a^	− .03	.02	− .03	− .08***
Attractiveness ^a^	.08*	.06	.04	.07*
Age × relationship status	− .09*	− .16***	.02	.01
Gender/sex × Attractiveness^a^	.07	.08*	.04	.03
Gender/sex × Masculinity^a^	− .13**	− .21***	− .14**	.04
Gender/sex × Sex. orientation	− .15	− .21*	− .03	− .03
Adj. R^2^	.081	.071	.090	.078

SDI-2 = Sexual Desire Inventory-2 (Spector et al., 1996); dyadic-P = dyadic (partner); dyadic-A = dyadic (attractive person). Multiple regression models (full models) are each based on *N* = 2744 observations. Participant gender/sex, relationship status, and sexual orientation were factor coded (0 = male, 1 = female; 0 = heterosexual, 1 = same-sex attracted; 0 = single, 1 = in a committed relationship). All continuous variables were z-standardized. Negative estimates indicate lower scores for women compared to men, for same-sex attracted compared to heterosexual people, and for people having a partner compared to singles. ^a^Self-rated. *p < .05, **p < .01, ***p < .001

Before describing the results in detail, we would like to note that the adjusted *R*^2^ of the models was very small (ranging from 0.071–0.090), indicating that little variance in sexual desire was explained. Female gender/sex negatively predicted dyadic desire (attractive person) and solitary desire. Age positively predicted dyadic sexual desire (partner). Of the self-perceptions, masculinity positively predicted both measures of dyadic sexual desire, while health negatively predicted solitary sexual desire and attractiveness was not predictive for any of the outcome measure. Having a partner positively predicted dyadic sexual desire (partner) and negatively predicted dyadic sexual desire (attractive person). In addition, having a partner negatively predicted solitary desire but this effect was only significant at *p* = 0.006. Finally, same-sex attraction positively predicted solitary sexual desire.

We found significant interaction effects, which are illustrated in Figs. 4 and 5: As indicated by the negative interaction of age and relationship status, the positive effect of having a partner on dyadic desire (partner) weakened with age. As indicated by the negative interaction of gender/sex and self-rated masculinity, the positive effect of self-rated masculinity on both types of dyadic desire was weaker for women compared to men. Of note, this effect was only significant at *p* < 0.001 in the case of dyadic desire (partner).

### Age Trends

In order to examine the effect of age on sexual desire in more detail, we conducted linear and polynomial trend analyses for all men (*n* = 2618) and women (*n* = 5532) separately. As before, the adjusted *R*^2^ indicated that all models explained only small amounts of variance (linear models: *R*^2^ ranging from − 0.000–0.015; polynomial models: *R*^2^ ranging from 0.002–0.026). Results of the linear trend analyses are shown in Table 19. For women, age was a positive significant predictor for solitary sexual desire, but unrelated to all other SDI-2 facets, when tested in a linear model. For men, age was a significant and positive predictor for all three SDI-2 facets except dyadic sexual desire (attractive person), when tested in a linear model.

**Fig. 2:**
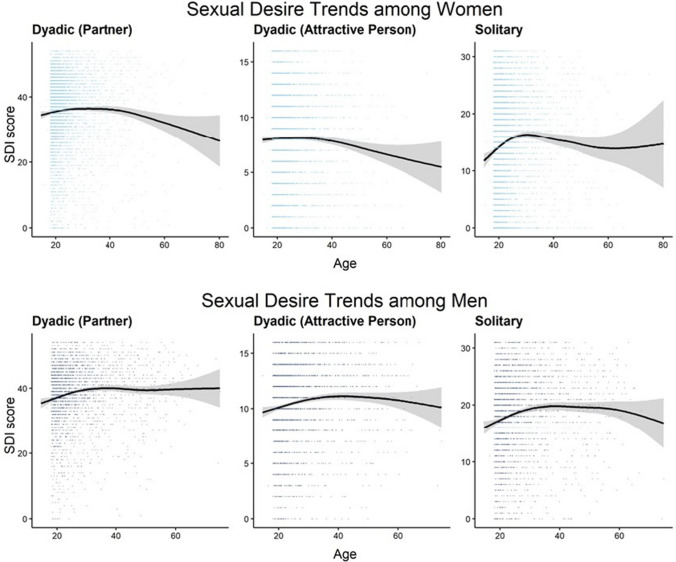
Age Trends in Sexual Desire. The upper and the lower panel show the sexual desire trends for women and men, respectively. The blue dots represent sexual desire scores of individuals with a given age. The black lines each represent the average sexual desire score regressed across individuals at different ages. The gray area depicts the corresponding 95% confidence bands

The results of the polynomial trend analyses can be observed from Table 3 and are illustrated in Fig. 2. For women, negative quadratic trends most consistently predicted their sexual desire from age across sexual desire types, yet this effect was only significant at *p* = 0.004 in the case of dyadic desire (attractive person). In the case of women’s solitary sexual desire, positive linear and cubic trends were also observed. Descriptively, age effects on individual differences in women’s dyadic sexual desire (both types) were marked by an initially positive effect that plateaued between the mid-twenties and mid-forties followed by a negative effect. In the case of women’s solitary desire, we observed a peak in the thirties, which was first followed by a negative effect and then by a slightly positive age effect after the age of 60. For men, positive linear, and negative quadratic trends significantly predicted sexual desire in the case of all sexual desire types. With the exception of dyadic sexual desire (attractive person), age effects on individual differences in men’s sexual desire were descriptively marked by a positive effect until age 40, by a slight negative effect followed by a positive effect around the age of 50, and a negative effect after age 60. Age effects on individual differences in men’s dyadic sexual desire (attractive person), in contrast, were descriptively marked by a positive effect until the late thirties or early forties and a negative effect thereafter.

**Table 3:** Polynomial trends of sexual desire among men and women

Trend	Men	Women
	SDI-2 score: Total	
Age	6.31***	1.95
Age^2^	− 5.36***	− 5.71***
Age^3^	1.41	2.62**
Adj. R^2^	.026	.007
	SDI-2 score: Dyadic-P	
Age	4.62***	0.22
Age^2^	− 3.63***	− 4.19***
Age^3^	2.27*	0.42
Adj. R^2^	.014	.003
	SDI-2 score: Dyadic-A	
Age	4.18***	− 2.00*
Age^2^	− 3.78***	− 2.86**
Age^3^	− 0.29	1.35
Adj. R^2^	.011	.002
	SDI-2 score: Solitary	
Age	5.17***	4.47***
Age^2^	− 4.59***	− 5.54***
Age^3^	0.38	4.08***
Adj. R^2^	.017	.012

SDI-2 = Sexual Desire Inventory-2 (Spector et al., 1996); dyadic-P = dyadic (partner); dyadic-A = dyadic (attractive person). Polynomial regression models are each based on the observation of *n* = 2618 men and *n* = 5532 women. All variables were z-standardized. *p < .05, **p < .01, ***p < .001

To explore possible interactions between gender/sex and age, we conducted additional trend analyses using the data of the whole sample (*N* = 8150), using gender/sex as a moderator. Thus, in addition to the linear, quadratic, and polynomial effects of age, we entered gender/sex and the interaction terms of gender/sex and each of the age effects into our models. Results were generally consistent with the gender-/sex-specific trend analyses reported above (see Table 20). Specifically, the additional analyses revealed three patterns: first, in addition to a negative effect of female gender/sex, positive linear and negative quadratic effects of age predicted sexual desire across all facets; second, the interaction between female gender/sex and the linear age effect was negatively associated with both facets of dyadic sexual desire, indicating that the positive linear effect of age on desire was less pronounced in women; third, and in contrast to the findings for dyadic sexual desire, solitary sexual desire was also predicted by a positive polynomial effect of age while none of the interaction terms was significant at *p* < 0.001.

### Group Comparisons by Gender/Sex and Sexual Orientation

**Fig. 3:**
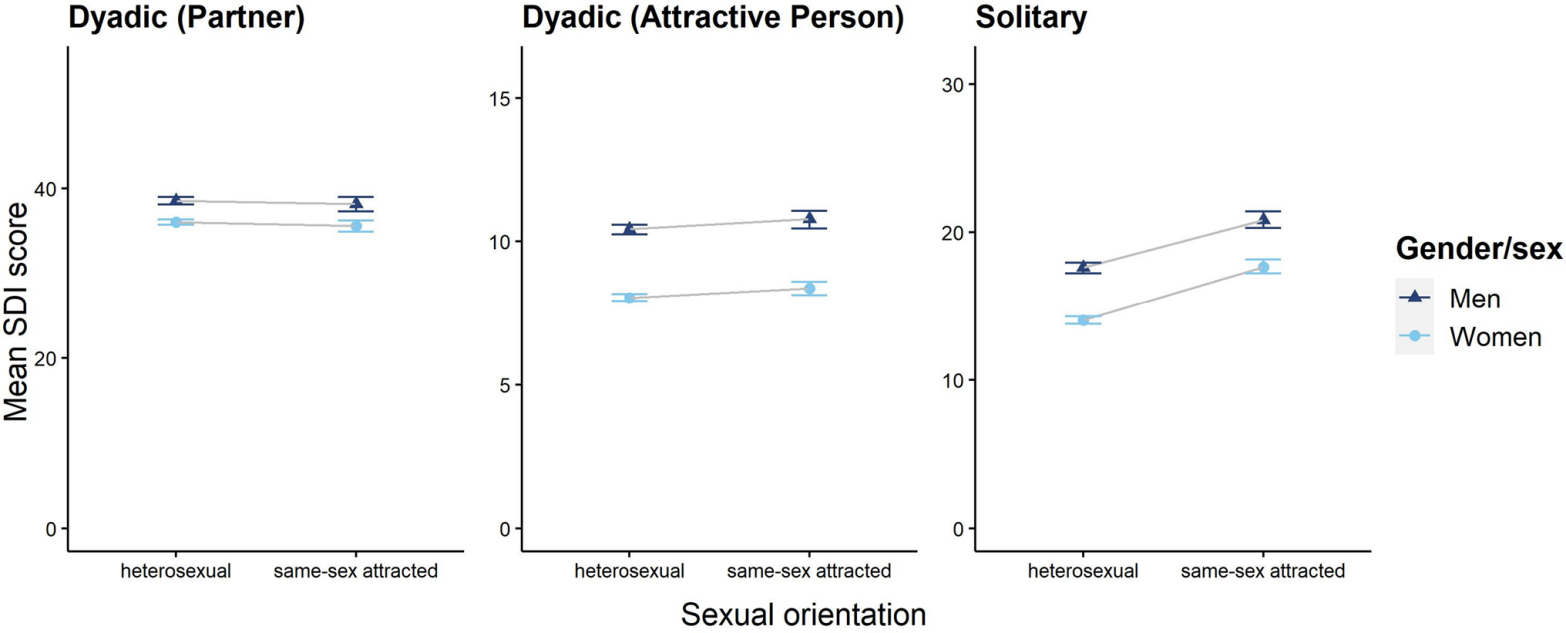
Interactions between Gender/Sex and Sexual Orientation. Means of sexual desire scores are shown with 95% confidence intervals as dots (women) and triangles (men). Please note that the gray lines were added for illustrative purposes, but do not represent data points

**Fig. 4:**
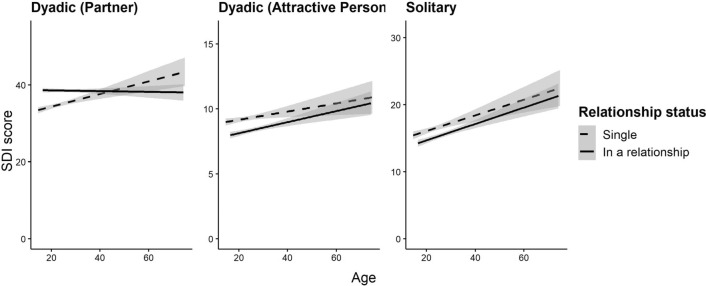
Interactions between Age and Relationship Status. The black lines each represent the simple slopes derived from the corresponding regression model (without control variables) for people without (dashed) and with (solid) a current romantic relationship. The gray area depicts the corresponding 95% confidence bands

**Fig. 5:**
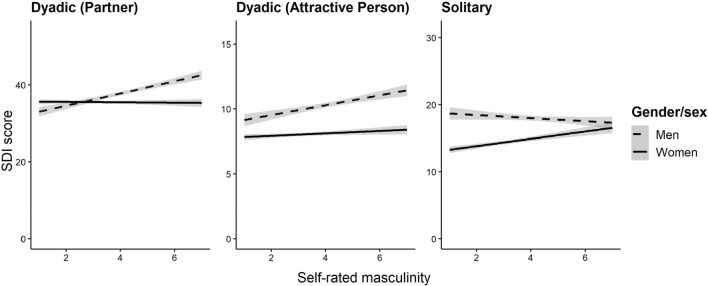
Interactions between Self-Rated Masculinity and Gender/Sex. The black lines each represent the simple slopes derived from the corresponding regression model (without control variables) for men (dashed) and women (solid). The gray area depicts the corresponding 95% confidence bands

After obtaining effects of gender/sex and sexual orientation on sexual desire in the full models, we examined these predictors by conducting group comparisons. Together, these findings are illustrated in Fig. 3.

The results of Welch t-tests comparing men’s with women’s sexual desire are shown in Table 4. Men’s sexual desire score was significantly higher across all facets, with small to medium effect sizes. Cohen’s *d* of the gender/sex effect was largest for dyadic sexual desire (attractive person), followed by solitary sexual desire, and smallest for dyadic sexual desire (partner). Bootstrapped comparisons based on 10 000 simulations each indicated that all of these differences across effect sizes were significantly different from each other.^3^

**Table 4:** Descriptive statistics, T-Test parameters and effect sizes (Cohen’s d) of the comparison of SDI-2 scores between men and women

SDI-2 score	*M* (*SD*)		*t*	*df*	Cohen’s *d*	95% CI
	Men	Women				
Total	71.75 (17.30)	61.89 (19.90)	22.86***	5837.3	0.52	[0.47, 0.56]
Dyadic-P	38.39 (9.78)	35.76 (11.07)	10.86***	5752.9	0.25	[0.20, 0.29]
Dyadic-A	10.50 (3.71)	8.05 (4.02)	27.05***	5522.3	0.62	[0.58, 0.67]
Solitary	18.38 (7.41)	14.76 (8.80)	19.35***	6011	0.43	[0.38, 0.48]

SDI-2 = Sexual Desire Inventory-2 (Spector et al., 1996); dyadic-P = dyadic (partner); dyadic-A = dyadic (attractive person). Means, standard deviations, and Welch t-tests for independent samples are each based on the observation of *n* = 2618 men and *n* = 5532 women. *** *p* < .001

The results of Welch t-tests comparing sexual desire scores between heterosexual and same-sex attracted men, as well as between heterosexual and same-sex attracted women are shown in Table 5. Looking at both men and women, individuals with same-sex attraction scored significantly higher on solitary sexual desire with a small effect.

**Table 5:** Descriptive statistics, T-Test parameters and effect sizes (Cohen’s d) of the comparison of SDI-2 scores between heterosexual and same-sex attracted individuals by gender/sex

SDI-2 score	*M* (*SD*)		*t*	*df*	Cohen’s *d*	95% CI
	Heterosexual	Same-sex attracted				
*Men*						
Total	71.05 (17.35)	74.44 (16.02)	4.19***	907.38	0.20	[0.10, 0.30]
Dyadic-P	38.56 (9.74)	38.17 (9.73)	− 0.81	849.24	− 0.04	[− 0.14, 0.06]
Dyadic-A	10.43 (3.71)	10.78 (3.55)	2.01*	879.95	0.10	[0.00, 0.19]
Solitary	17.62 (7.51)	20.84 (6.50)	9.63***	963.31	0.44	[0.34, 0.54]
*Women*						
Total	61.42 (19.19)	65.23 (20.57)	5.47***	1604.8	0.20	[0.13, 0.26]
Dyadic-P	36.05 (10.59)	35.59 (11.63)	− 1.18	1578.3	− 0.04	[− 0.11, 0.02]
Dyadic-A	8.04 (3.97)	8.37 (4.04)	2.42*	1667.0	0.08	[0.02, 0.15]
Solitary	14.07 (8.76)	17.67 (8.29)	12.51***	1763.9	0.42	[0.35, 0.48]

SDI-2 = Sexual Desire Inventory-2 (Spector et al., 1996). Means, standard deviations, and Welch t-tests for independent samples are based on the comparisons between *N* = 524 same-sex attracted and *N* = 1815 heterosexual men, and between *N* = 1076 same-sex attracted and *N* = 4067 heterosexual women. *p < .05, **p < .01, ***p < .001

### Robustness Check

In our sample, age was not evenly distributed, such that only few participants under age 18 (*n* = 8) or above age 60 (*n* = 38) were included. In order to test whether the data from these participants biased the results, we repeated our analyses with an age-truncated sample, excluding participants under age 18 and over age 60 (*n* = 46; *N*_truncated_ = 8104) as a robustness check. Results from these analyses can be obtained from the online supplement at https://osf.io/jk5zh. Most results from the main analyses were unaffected, but some deviations occurred: In women, the negative quadratic trend (β =  − 3.15, *p* = 0.002) predicting dyadic sexual desire (partner) and the cubic trend (β = 2.72, *p* = 0.006) predicting solitary sexual desire were no longer significant at *p* < 0.001. In men, the negative quadratic trend (β =  − 2.68, *p* = 0.007) predicting dyadic sexual desire (attractive person) and the negative quadratic trend (β =  − 3.23, *p* = 0.001) predicting solitary sexual desire were no longer significant at *p* < 0.001. Despite these (mainly subtle) differences in the level of significance, the direction of trends remained the same.

## Discussion

Using a large online sample, covering a broad age range, we examined effects of age, gender/sex, sexual orientation, relationship status, and self-rated masculinity, attractiveness, and health on sexual desire. Our findings add to the literature on sexual desire in three important ways: First, trend analyses revealed nonlinear associations between age and sexual desire for both men and women, while differences across genders/sexes and sexual desire facets became apparent. Second, average sexual desire was higher among men. Going beyond previous research, we found that the gender/sex difference regarding dyadic sexual desire (partner) was smaller than the difference regarding the other desire facets. Third, remarkably little variance in sexual desire was explained despite the large number of predictors, pointing to the impact of unknown variables. Below we review the effects of each predictor in relation to dyadic (partner), dyadic (attractive other), and solitary sexual desire.

### Age Effects

Contrary to our prediction (Hypothesis 1a) and other studies on sexual desire (e.g., Beutel et al., 2008), we did not find a general (linear) negative association between age and sexual desire when examined across men and women. Instead, results are consistent with the studies that found the most pronounced declines of sexual desire starting at higher age (i.e., around age 60; Burghardt et al., 2020; DeLamater & Sill, 2005). Moreover, our findings might reflect a more complex, nonlinear relationship between age and different forms of sexual desire that varies by gender/sex. Given the small effect sizes, however, these findings have to be regarded with caution and further replication attempts should be initiated.

Partly supporting our prediction that women’s sexual desire would show a zenith in mid-adulthood (Hypothesis 1b), we found significant negative quadratic age trends predicting all forms of women’s sexual desire. However, patterns did not exactly meet our age predictions: Whereas women’s dyadic sexual desire was positively associated with age until the mid-twenties, it was at a similar level for women between their mid-twenties and -forties, rather than being highest among women in their early thirties as predicted by evolutionary theories (Easton et al., 2010; Schmitt et al., 2002). Still, this pattern is compatible with the hypothesis that women’s dyadic sexual desire is heightened during the life phase where women are most fertile. In addition, it could be argued that women’s dyadic sexual desire is influenced by family planning and is highest at ages where many women wish to become mothers (Levine, 2003). In contrast, women’s solitary sexual desire was highest among women in their mid-thirties, negatively associated with age afterward, but again positively associated with age after age 60. Consistent with evolutionary hypotheses, this might be understood in the way that women's solitary sexual desire is less affected by age-related biological changes than their dyadic sexual desire. Since solitary sexual activity does not directly relate to reproduction as partnered sex, women’s solitary sexual desire might fulfill a different, less age- and fertility-related function than their dyadic sexual desire. In addition to these biological factors, solitary sexual desire might be less affected than dyadic sexual desire by the loss of a partner, which becomes more likely as people age and is more likely for women compared to men (Kontula & Haavio-Mannila, 2009).

In contrast to our prediction (Hypothesis 1c), age was neither negatively nor purely linearly associated with male participants’ sexual desire: Trend analyses revealed that men’s sexual desire followed both a positive linear and a negative quadratic trend across the different facets of sexual desire. On average, men’s sexual desire was positively associated with age until age 40 and illustrated a more complex pattern afterward. Dyadic (partner) and solitary desire were at a similar level for men aged between 40 and 60. In contrast, dyadic sexual desire (attractive person) was negatively associated with age after the age 40. This age effect on sexual desire relating to an attractive person might reflect this shift in social needs: According to socioemotional selectivity theory (Carstensen, 1991), most people have a strong motive for exploration and novel experiences when they are young, but an increasing need for close and familiar relationships after midlife. Thus, after age 40, dyadic desire of most men might focus on sex with a committed romantic partner instead of sex with unfamiliar people.

In sum, our results on age effects point to both similarities and differences between men and women. First, overall, effects were very small, pointing to a subordinate role of age in explaining individual differences in sexual desire within our sample. Second, as reflected by the negative quadratic effects in our trend analyses, sexual desire might profit from increases in sexual experience (Hally & Pollack, 1993; Impett & Tolman, 2006) in both gender/sexes, until other factors, such as losses of sexual and relationship functioning or declines in health, cancel these gains (also see Forbes et al., 2017). Thus, challenging common assumptions (e.g., Barr et al., 2002; Basson, 2000; Baumeister, 2000), men’s desire, similar to women’s, might be influenced by a number of life circumstances instead of being only driven by biological factors. At the same time, our results pointed to gender/sex specificities in age effects on sexual desire: First, compared to women, men might maintain higher sexual desire until higher ages. Second, the associations between women’s age and their dyadic and solitary desire might be relatively independent from each other, but more parallel in men.

### Gender/Sex Effects

As expected (Hypothesis 2a) and consistent with the large body of previous studies (e.g., Baumeister et al., 2001; Hyde, 2005), men reported higher dyadic and solitary sexual desire on average than women. Notably, this effect was smaller for dyadic sexual desire (partner) compared to all other facets of sexual desire. Even though we based our prediction on the original dyadic desire instead of the more specific dyadic desire (partner) scale, this finding supports our prediction that men and women’s dyadic desire would be more similar than their solitary desire (Hypothesis 2b). Our results add to the understanding of gender/sex differences in trait sexual desire, by showing that difference in dyadic desire might be mainly driven by desire for attractive others as opposed to desire for a partner.

In sexual strategies theory (Buss & Schmitt, 1993; Gangestad & Simpson, 2000), relatively high sexual dyadic desire (partner) in women could be understood as an adaptation supporting the goal to find and reproduce with long-term mates, while the higher dyadic desire (attractive person) in men could serve the goal to reproduce with more short-term mates. While evolutionary theory holds little explanation for the gender/sex effects on solitary sexual desire, both higher solitary and dyadic (attractive person) desire in men, as well as relative gender/sex similarity in dyadic desire (partner) could be explained from social learning theory (Bandura, 1986; Bussey & Bandura, 1999). For women, sexual desire relating to a partner, compared to desire for masturbation and sexual activity outside the relationship context, might be more accepted in society, easier to observe, and more reinforced. Finally, findings can be interpreted in the context of social structural theory (Eagly & Wood, 1999). While the higher societal status of men compared to women is often replicated in (heterosexual) couple dynamics (Knudson‐Martin, 2013), the greater gender/sex similarity in dyadic sexual (partner) desire might reflect smaller power discrepancies between genders/sexes within close relationships compared to other social contexts.

### Sexual Orientation Effects

Our prediction regarding the link between sexual orientation and sexual desire (Hypothesis 3) was partly supported: same-sex attracted women reported higher levels of solitary sexual desire than heterosexual women and, contrasting with our prediction, the same was found for men; the difference between same gender/sex attracted and heterosexual individuals did not hold for dyadic sexual desire in either gender/sex. These findings correspond with other studies that found higher level of solitary sexual desire in non-heterosexual compared to (exclusively) heterosexual participants (Lorenz, 2019; Peixoto, 2019). Notably, Peixoto found higher dyadic desire relating to an attractive other in gay compared to heterosexual men, but this finding did not replicate in our study. Therefore, results suggest that same-sex attraction positively relates to solitary sexual desire, regardless of gender/sex. One possible explanation for this finding is that same-sex attracted individuals might spend more time reflecting their own sexuality and are therefore more aware of their solitary desire. Another possibility is that same-sex attracted people might be more used to non-compliance with social norms than heterosexual ones and therefore less inhibited in reporting solitary desire in spite of the fact that masturbation is still stigmatized by society. Finally, in comparison to hetero- and bisexual people, same-sex attracted individuals (especially when not living in big cities) have access to a smaller mating pool. If this is true, they might engage in more solitary sexual activities (see Persson et al., 2016) and therefore cultivate higher solitary desire. Future research on sexual orientation effects on sexual desire and how this relates to dyadic and solitary sexual behavior is required.

### Self-Rated Masculinity Effects

In line with our prediction (Hypothesis 4a), dyadic sexual desire (both types) was positively related to self-rated masculinity, even though the effect on dyadic desire (attractive person) was not significant below *p* = 0.001. However, no effect was found for solitary sexual desire. Since men usually report higher solitary sexual desire and that corresponding behavior is more typical and accepted for men than women (Petersen & Hyde, 2011, masculinity could be expected to have a greater effect on solitary than dyadic sexual desire. Instead, our findings suggest a social role of self-rated masculinity in the context of desire for a sexual partner. In addition, self-rated masculinity did not have a stronger effect on female compared to male sexual desire, as we had expected (Hypothesis 4b). Instead, the opposite was true: While no interaction effect occurred in the case of solitary sexual desire, the effect of self-rated masculinity on dyadic desire was stronger for men. Thus, perceiving oneself as masculine within a dyadic sexual context might be mainly relevant for men’s positive sexual experience.

### Relationship Status Effects

As predicted (Hypothesis 5a), having a partner positively predicted dyadic sexual desire (partner) and negatively predicted solitary sexual desire, even though this effect was not significant below *p* = 0.001. In addition, we found a negative effect on dyadic desire (attractive person). Our results highlight the potential effect of life circumstances on sexual desire: People’s sexual desire might adapt to the given possibilities to act out their desired sexual activities, with higher dyadic sexual desire relating to a partner when a partner is also available, and higher dyadic desire relating to an attractive person and higher solitary desire when not. Alternatively, people with higher dyadic desire (partner) might be more likely to enter and stay in a romantic relationship. Contrasting with our prediction (Hypothesis 5b; c.f. Kontula & Haavio-Mannila, 2009), we found that the positive effect of having a partner on dyadic desire (partner) was weaker for older individuals. Since age was not evenly distributed across the sample, such that only few older individuals without a partner (15 out of 65 adults over age 50) participated, however, power of this interaction effect was probably low and the finding should be treated with caution. As an alternative explanation, the negative interaction might reflect that age is confounded with relationship duration, which was associated with lower sexual desire (Dawson & Chivers, 2014; Murray & Milhausen, 2012). Because relationship duration was not measured in this study, we could not examine this possibility.

### Other Effects

Somewhat surprising, self-rated health did not relate to any of the dyadic sexual desire facets and negatively predicted solitary sexual desire (c.f., Mitchell et al., 2013). It could be speculated that these findings reflect that poor health affects the ability to have partnered sex and that sexual activity outside an interpersonal context is more attractive for individuals not feeling well. However, we did not find a positive effect of health on dyadic desire, adding no support to the idea that health might set preferences regarding dyadic versus solitary sexual activity. Finding little health effects overall might be due to the fact that health was assessed in relation to the participants’ perception of other people’s health. This way, it is unclear who the participants referred to and what their actual, objective health status was. Nonetheless, the measure should reflect subjective well-being to some degree.

While self-rated attractiveness was positively related to most types of sexual desire, it did not predict any outcomes in the full models. This is surprising since previous studies found positive effects of related variables, such as sexual self-esteem (Kontula & Haavio-Mannila, 2009) and positive body image (Koch et al., 2005; Seal et al., 2009). Therefore, we assume shared variance with other predictors of sexual desire included in this study: In both genders/sexes, self-rated attractiveness was related to health and, in men, it was additionally related to masculinity. The lack of self-rated attractiveness effects could not be explained by gender/sex differences either: Descriptively, self-rated attractiveness had a more pronounced effect on female sexual desire, but these effects were small and not significant.

### Strengths and Limitations

The current study had several strengths. First, we pursued a high degree of transparency and reduced researcher degrees of freedom (e.g., Nosek et al., 2015; Wicherts et al., 2016) by preregistering our hypotheses and analyses. Second, our sample was large, enabling us to test our hypotheses with sufficient power, and relatively diverse with regard to age and different sexual orientations. Third, the use of nonlinear trend analyses shed new light on previously oversimplified age effects on men and women’s sexual desire. Finally, by differentiating across dyadic (partner), dyadic (attractive person), and solitary sexual desire, we provide nuanced insights into different aspects of sexual desire that match the factor structure of our data and keep up with current standards on sexual desire measured with the SDI-2 (e.g., Mark et al., 2018; Moyano et al., 2017; Vallejo-Medina et al., 2020).

Our study also had some limitations. First, the cross-sectional design of the study cannot address age effects on sexual desire that may be due to cohort effects. In a study covering the years 2000–2018, Ueda et al. (2020) found that sexual inactivity increased among young adults. Similarly, age effects on sexual desire found in this study might reflect that participants in the mid-age range show higher sexual desire than those born before or after independent from age. Therefore, it would be helpful to track individual trajectories of age-associated changes in sexual desire in a longitudinal design.

Second and related to the first limitation, the correlational nature of our data did not allow for causal conclusions, even though some directions of influence (i.e., influence from biological and psychosocial predictors on sexual desire) seem more likely than others. By examining the effects of previously assessed variables on sexual desire at later measurements, a longitudinal study design would address this limitation, too.

A third limitation pertains to the exclusive use of self-reports. Self-reports can be biased by effects of social desirability. For example, women are known to underreport sexual motivation, thoughts, and behavior more frequently than men (Petersen & Hyde, 2011). At the same time, self-reports are the best way to assess an individual’s subjective experience and therefore essential for the assessment of sexual desire. Future studies could include measures of social desirability (e.g., Stöber, 2001) to control for potential biases.

Finally, very little variance was explained in our analyses. As a possible explanation, there are numerous variables potentially relevant to sexual desire, such as frequency of sexual intercourse (Kontula & Haavio-Mannila, 2009) or relationship satisfaction (Brezsnyak & Whisman, 2004; Davies et al., 1999) that remained unconsidered. In addition, many of the included variables might have been assessed in too unspecific ways to explain individual differences in sexual desire. For example, relationship status did not inform about relationship length, a factor that has been negatively associated with sexual desire in previous research (Dawson & Chivers, 2014; Murray & Milhausen, 2012). Similarly, single items have proven as useful measures for self-rated masculinity, attractiveness, and health when studying mate preferences (e.g., Feinberg et al., 2012; Kandrik & DeBruine, 2012), but are likely insufficient to capture more complex aspects of these constructs that might relate to sexual desire. In future studies on sexual desire, more and more detailed measures of variables relevant to a person’s romantic relationships and identity should be included.

### Concluding Remarks

Sexual desire represents an essential component of human sexuality, but relatively little is known about the exact nature of age effects on sexual desire. In this study, we found evidence for both differences and similarities between the sexual desire of men and women and corresponding age effects. While men showed higher sexual desire levels on average, sexual desire was highest among middle-aged individuals in both genders/sexes. Generally, study results are compatible with the hypothesis that women experience their highest levels of sexual desire during fertile years, (Easton et al., 2010; Schmitt et al., 2002). Furthermore, they suggest that both men and women’s sexual desire levels and corresponding age effects relate to a wide array of biological and psychosocial variables. Facing the small portions of variance explained, it is suggested that additional variables, that were not included in the study—such as relationship length (Dawson & Chivers, 2014) and satisfaction (Davies et al., 1999)—might play an important role as well. In these terms, women’s and men’s sexual desire may be more similar than many commonly believe. Differentiating between dyadic sexual desire relating to a partner and to an attractive person provided valuable and nuanced insights into effects of age, gender/sex, and relationship status on sexual desire. Therefore, we recommend that researchers measuring sexual desire with the SDI-2 should consider using the facets suggested by Moyano et al. (2017). Moreover, future studies should assess sexual desire longitudinally and include relationship-specific measures, as well as more nuanced measures of health and aspects relating to a person’s self-concept.

## Data Availability

On our OSF page (https://osf.io/rba2x), we publish all data necessary to reproduce reported results and provide scripts for all data analyses reported in this manuscript. In addition, we share a complete list with item wordings and response formats used in the current study at OSF.

## Appendix A

Originally, Spector et al. (1996) conceptualized the SDI-2 with two subfacets named dyadic sexual desire and solitary sexual desire. In a recent study however, Moyano et al. (2017) recommend further splitting the dyadic sexual desire scale, resulting in three subscales of the questionnaire. Even though we had already preregistered our hypotheses and conducted the corresponding analyses based on the original facets by Spector and colleagues, we wished to examine which model—two or three factors—fitted our data best before proceeding to hypothesis testing. Table 6 provides an overview over the SDI-2 items and their assignment to the original scales (Spector et al., 1996) or the scales suggested by Moyano et al. (2017), using a two-factor or a three-factor structure, respectively.

In order to determine, which of these structures provides a better fit for our data, we conducted a set of confirmatory factor analyses (CFAs) using the R package lavaan (Rosseel, 2012). Scale reliability measures were calculated using the package userfriendlyscience (Peters, 2014). In CFA, a critical concern is whether factors represent meaningful, distinguishable constructs or method effects (Marsh, 1996): While previous research (e.g., Mark et al., 2018) conceptualizes dyadic desire (partner) and dyadic desire (attractive person) as substantial factors, distinguishable components in CFA might simply reflect the fact that these items share common wording (i.e., “a partner” versus “an attractive person”). To investigate this possibility, we conducted total of four CFAs. First, we specified two simple models defining either a two- or three-factor structure. Second, for each of these factor solutions, we specified an additional model, which included autocorrelations of the items sharing common wording in order to represent potential method effects(see Marsh, 1996). The fit indices of all four models are shown in Table 7. As can be seen, model fit indices improved (a) by including autocorrelations and (b) by distinguishing between three instead of two factors. Factor loadings and internal consistencies of the two-factor and three-factor solutions including autocorrelations of items with shared wording can be found in Tables 8 and 9. Comparing both of these models, likelihood ratio test indicated a significantly better fit for the model with a three-factor structure compared to the model with a two-factor structure (*χ*^2^_Diff_ (2) = 389.04, *p* < 0.001). Thus, even after controlling for a method effect, our results suggested that a differentiation between two different dyadic facets—dyadic desire (partner) and dyadic desire (attractive person)—and solitary desire, resulting in a total of three sexual desire facets, would be more appropriate for analyzing our data.

**Table 6:** Items and descriptive statistics of the SDI-2

Factors and Items	*M* (*SD*)
*Dyadic desire*	
1. During the last month, how often would you have liked to engage in sexual activity with a partner (for example, touching each other’s genitals, giving or receiving oral stimulation, intercourse, etc.)? ^P^	4.77 (1.88)
2. During the last month, how often have you had sexual thoughts involving a partner? ^P^	4.71 (1.98)
3. When you have sexual thoughts, how strong is your desire to engage in sexual behavior with a partner? ^P^	5.71 (1.87)
4. When you first see an attractive person, how strong is your sexual desire? ^A^	4.12 (2.21)
5. When you spend time with an attractive person (for example, at work or school), how strong is your sexual desire? ^A^	4.72 (2.19)
6. When you are in romantic situations (such as candle-lit dinner, a walk on the beach, etc.), how strong is your sexual desire? ^P^	5.35 (2.06)
7. How strong is your desire to engage in sexual activity with a partner? ^P^	6.12 (1.94)
8. How important is it for you to fulfill your sexual desire through activity with a partner? ^P^	5.24 (2.30)
9. Compared to other people of your age and sex, how would you rate your desire to behave sexually with a partner? ^P^	4.70 (2.11)
*Solitary desire*	
10. During the last month, how often would you have liked to behave sexually by yourself (for example, masturbating, touching your genitals, etc.)?	3.78 (2.22)
11. How strong is your desire to engage in sexual behavior by yourself?	4.18 (2.39)
12. How important is it for you to fulfill your desires to behave sexually by yourself?	3.92 (2.62)
13. Compared to other people of your age and sex, how would you rate your desire to behave sexually by yourself?	4.04 (2.35)
*No factor (item adds to total desire only)*	
14. How long could you go comfortably without having sexual activity of some kind?	3.70 (1.90)

SDI-2 = Sexual Desire Inventory-2 (Spector et al., 1996); Means and standard deviations are based on *N* = 8150 observations. The factors indicated in the rows resemble the original structure with two factors. For each of the dyadic items, upper case letters indicate to which of the scales it is assigned when applying the three-factor structure (P = partner; A = attractive person)

**Table 7:** Model fits of different factor solutions with the SDI-2 items

Model	Model fit					
	*χ*^*2*^	*Df*	CFI	TLI	RMSEA	SRMR
1. Two factors, no autocorrelation	8664.475***	64	.863	.833	.128	.078
2. Two factors, autocorrelation	2921.760***	58	.954	.939	.078	.061
3. Three factors, no autocorrelation	3993.944***	62	.937	.921	.088	.049
4. Three factors, autocorrelation	2532.723***	56	.961	.945	.074	.046

SDI-2 = Sexual Desire Inventory-2 (Spector et al., 1996); Confirmatory factor analysis (CFA) was based on *N* = 8150 observations and estimated using maximum likelihood method. *** *p* < .001

**Table 8:** Factor loadings and internal consistencies of the two-factor solution with the SDI-2 items

Factors and items	Loadings (SE)	Stand. loadings	α	ω	*GLB*
*Dyadic desire*			.88	.88	.88
1	1.31 (.02)	0.70			
2	1.26 (.02)	0.64			
3	1.42 (.02)	0.76			
4	1.13 (.02)	0.51			
5	1.14 (.02)	0.52			
6	1.16 (.02)	0.56			
7	1.67 (.02)	0.86			
8	1.57 (.02)	0.68			
9	1.66 (.02)	0.79			
*Solitary desire*			.91	.92	.93
10	1.71 (.02)	0.77			
11	2.19 (.02)	0.92			
12	2.26 (.02)	0.86			
13	2.04 (.02)	0.86			

SDI-2 = Sexual Desire Inventory-2 (Spector et al., 1996). The confirmatory factor analysis was based on *N* = 8150 observations and estimated using maximum likelihood method. Item 14 was not included in the model because it is a part of the total desire score but not of one of the desire subscales. Internal consistencies are given as Cronbachs’s α, total omega ω, and greatest lower bound (GLB)

See Tables 6, 7, 8, and 9.

**Table 9:** Factor loadings and internal consistencies of the three-factor solution with the SDI-2 items

Factors and items	Loadings (SE)	Stand. loadings	α	ω	GLB
*Dyadic desire *(partner)			.88	.88	.89
1	1.32 (.02)	0.70			
2	1.26 (.02)	0.64			
3	1.42 (.02)	0.76			
6	1.15 (.02)	0.56			
7	1.69 (.02)	0.87			
8	1.59 (.02)	0.69			
9	1.67 (.02)	0.79			
*Dyadic desire (attractive person)*			.84	−	−
4	1.87 (.02)	0.85			
5	1.88 (.02)	0.86			
*Solitary desire*			.91	.92	.93
10	1.71 (.02)	0.77			
11	2.19 (.02)	0.92			
12	2.26 (.02)	0.86			
13	2.04 (.02)	0.86			

SDI-2 = Sexual Desire Inventory-2 (Spector et al., 1996). The confirmatory factor analysis was based on *N* = 8150 observations and estimated using maximum likelihood method. Item 14 was not included in the model because it is a part of the total desire score but not of one of the desire subscales. Internal consistencies are given as Cronbachs’s α, total omega ω, and greatest lower bound (GLB). For the dyadic desire (attractive person) scale, no ω and GLB could be calculated because it consists of too few items

## Appendix B

In the following, we report all results from the main article including the original dyadic desire scale (Spector et al., 1996).

See Tables 10, 11, 12, 13 and 14.

**Table 10:** Descriptive statistics and intercorrelations of the continuous study variables by gender/sex

	*M*	*SD*	Intercorrelations								*N*
			1	2	3	4	5	6	7	8	
*Men*											
1. Age	27.34	9.48									2618
2. SDI: Total	71.75	17.30	**.12**								2618
3. SDI: Dyadic	48.89	12.17	**.10**	**.90**							2618
4. SDI: Dyadic-P	38.39	9.78	**.09**	**.85**	**.97**						2618
5. SDI: Dyadic-A	10.50	3.71	**.08**	**.69**	**.73**	**.53**					2618
6. SDI: Solitary	18.38	7.41	**.10**	**.72**	**.35**	**.30**	**.34**				2618
7. Attractiveness^a^	4.62	1.29	− .02	**.10**	**.14**	**.14**	**.09**	− .01			1525
8. Masculinity^a^	4.12	1.34	**.13**	**.14**	**.22**	**.22**	**.14**	− .04	**.31**		1525
9. Health^a^	4.65	1.41	.02	.02	.08	.08	.05	** − .10**	**.42**	**.33**	1239
*Women*											
1. Age	23.74	6.75									5532
2. SDI: Total	61.89	19.90	.03								5532
3. SDI: Dyadic	43.81	13.37	− .01	**.90**							5532
4. SDI: Dyadic-P	35.76	11.07	− .00	**.86**	**.96**						5532
5. SDI: Dyadic-A	8.05	4.02	− .03	**.62**	**.67**	**.45**					5532
6. SDI: Solitary	14.76	8.80	**.06**	**.76**	**.42**	**.38**	.**33**				5532
7. Attractiveness^a^	4.48	1.29	.04	**.17**	**.18**	**.17**	**.12**	**.08**			3530
8. Masculinity^a^	3.08	1.53	.02	.05	.01	− .01	.04	**.09**	** − .06**		3510
9. Health^a^	4.17	1.34	.02	.01	**.06**	**.06**	.03	** − .06**	**.36**	− .04	3168

SDI-2 = Sexual Desire Inventory-2 (Spector et al., 1996); dyadic-P = dyadic (partner); dyadic-A = dyadic (attractive person). All measures were assessed via self-reports. Correlations in bold font are significant (p < .001, two-tailed). ^a^ Self-rated

**Table 11:** Coefficients of the multiple regression models (full models) predicting the SDI-2 scores

	SDI-2 score				
Predictor	Total	Dyadic	Dyadic-P	Dyadic-A	Solitary
Age	.10**	.09**	.11***	− .00	.09**
Gender/sex	–.31***	–.20***	–.07	− .46***	− .28***
Sexual orientation	.31***	.16*	.15	.13	.42***
Masculinity^a^	.18***	.22***	.21***	.17***	.06
Relationship status	.13***	.22***	.33***	− .16***	− .11**
Health ^a^	− .03	.01	.02	− .03	− .08***
Attractiveness^a^	.08*	.07*	.06	.04	.07*
Age × relationship status	− .09*	− .13**	− .16***	.02	.01
Gender/sex × Attractiveness^a^	.07	.08	.08*	.04	.03
Gender/sex × Masculinity ^a^	− .13**	− .22***	− .21***	− .14**	.04
Gender/sex × Sex. orientation	− .15	− .18	− .21*	− .03	− .03
Adj. R^2^	.081	.071	.071	.090	.078

SDI-2 = Sexual Desire Inventory-2 (Spector et al., 1996); dyadic-P = dyadic (partner); dyadic-A = dyadic (attractive person). Multiple regression models (full models) are each based on *N* = 2744 observations. Participant gender, relationship status, and sexual orientation were factor coded (0 = male, 1 = female; 0 = heterosexual, 1 = same-sex attracted; 0 = single, 1 = in a committed relationship). All continuous variables were z-standardized. Negative estimates indicate lower scores for women compared to men, for same-sex attracted compared to heterosexual people, and for people having a partner compared to singles. ^a^Self-rated. **p* < .05, ***p* < .01, ****p* < .001

**Table 12:** Polynomial trends of sexual desire among men and women

Trend	Men	Women
	SDI-2 score: Total	
Age	6.31***	1.95
Age^2^	− 5.36***	− 5.71***
Age^3^	1.41	2.62**
Adj. R^2^	.026	.007
	SDI-2 score: Dyadic	
Age	4.99***	− 0.42
Age^2^	− 4.07***	− 4.33***
Age^3^	1.82	0.75
Adj. R^2^	.016	.003
	SDI-2 score: Dyadic-P	
Age	4.62***	0.22
Age^2^	− 3.63***	− 4.19***
Age^3^	2.27*	0.42
Adj. R^2^	.014	.003
	SDI-2 score: Dyadic-A	
Age	4.18***	− 2.00*
Age^2^	− 3.78***	− 2.86**
Age^3^	− 0.29	1.35
Adj. R^2^	.011	.002
	SDI-2 score: Solitary	
Age	5.17***	4.47***
Age^2^	− 4.59***	− 5.54***
Age^3^	0.38	4.08***
Adj. R^2^	.017	.012

SDI-2 = Sexual Desire Inventory-2 (Spector et al., 1996); dyadic-P = dyadic (partner); dyadic-A = dyadic (attractive person). Polynomial regression models are each based on the observation of *n* = 2618 men and *n* = 5532 women. All variables were z-standardized. **p* < .05, ***p* < .01, ****p* < .001

**Table 13:** Descriptive statistics, T-Test parameters and effect sizes (Cohen’s d) of the comparison of SDI-2 scores between men and women

SDI-2 score	*M* (*SD*)		*t*	*df*	Cohen’s *d*	95% CI
	Men	Women				
Total	71.75 (17.30)	61.89 (19.90)	22.86***	5837.3	0.52	[0.47, 0.56]
Dyadic	48.88 (12.17)	43.81 (13.37)	17.03***	5597.8	0.39	[0.34, 0.44]
Dyadic-P	38.39 (9.78)	35.76 (11.07)	10.86***	5752.9	0.25	[0.20, 0.29]
Dyadic-A	10.50 (3.71)	8.05 (4.02)	27.05***	5522.3	0.62	[0.58, 0.67]
Solitary	18.38 (7.41)	14.76 (8.80)	19.35***	6011	0.43	[0.38, 0.48]

SDI-2 = Sexual Desire Inventory-2 (Spector et al., 1996); dyadic-P = dyadic (partner); dyadic-A = dyadic (attractive person). Means, standard deviations, and Welch t-tests for independent samples are each based on the observation of *n* = 2618 men and *n* = 5532 women. *** *p* < .001

**Table 14:** Descriptive statistics, T-test parameters and effect sizes (Cohen’s d) of the comparison of SDI-2 scores between heterosexual and same-sex attracted individuals by gender/sex

SDI-2 score	*M* (*SD*)		*t*	*df*	Cohen’s *d*	95% CI
	Heterosexual	Same-sex attracted				
*Men*						
Total	71.05 (17.35)	74.44 (16.02)	4.19***	907.38	0.20	[0.10, 0.30]
Dyadic	48.99 (12.14)	48.96 (11.87)	− 0.05	863.84	− 0.00	[− 0.10, 0.09]
Dyadic-P	38.56 (9.74)	38.17 (9.73)	− 0.81	849.24	− 0.04	[− 0.14, 0.06]
Dyadic-A	10.43 (3.71)	10.78 (3.55)	2.01*	879.95	0.10	[0.00, 0.19]
Solitary	17.62 (7.51)	20.84 (6.50)	9.63***	963.31	0.44	[0.34, 0.54]
*Women*						
Total	61.42 (19.19)	65.23 (20.57)	5.47***	1604.8	0.20	[0.13, 0.26]
Dyadic	44.09 (12.77)	43.96 (14.07)	− 0.27	1575	− 0.01	[− 0.08, 0.06]
Dyadic-P	36.05 (10.59)	35.59 (11.63)	− 1.18	1578.3	− 0.04	[− 0.11, 0.02]
Dyadic-A	8.04 (3.97)	8.37 (4.04)	2.42*	1667.0	0.08	[0.02, 0.15]
Solitary	14.07 (8.76)	17.67 (8.29)	12.51***	1763.9	0.42	[0.35, 0.48]

SDI-2 = Sexual Desire Inventory-2 (Spector et al., 1996). Means, standard deviations, and Welch t-tests for independent samples are based on the comparisons between *N* = 524 same-sex attracted and *N* = 1815 heterosexual men, and between *N* = 1076 same-sex attracted and *N* = 4067 heterosexual women

^*^*p* < .05, ****p* < .001

## Appendix C

In our preregistration, we conducted a number of analyses testing hypotheses, which are not presented in the main article. First, we hypothesized that men would perceive their own attractiveness more positively than women. Second, we expected that self-rated attractiveness would partly explain the gender/sex difference in sexual desire (partial mediation). Likewise, we expected that self-rated masculinity would partly explain the gender/sex difference in sexual desire (partial mediation). Finally, we conducted exploratory analyses testing whether hormonal contraception affects the sexual desire of women prior menopause. In the following, we present the results of the tests corresponding to each of these hypotheses for all sexual desire scales (i.e., total, dyadic, dyadic (partner), dyadic (attractive person), and solitary sexual desire).

See Tables 15, 16 and 17.

**Table 15:** Coefficients of the mediation analyses: gender/sex–self-rated attractiveness–sexual desire and sobel tests (Z-values)

Step 1: Regression of gender/sex on self-rated attractiveness: − .11***					
Step 2–4: Regressions on sexual desire					
Predictor	SDI-2 score				
	Total	Dyadic	Dyadic-P	Dyadic-A	Solitary
Step 2: Gender/sex	− .48***	− .36***	− .23***	− .57***	− .41***
Step 3–4: Gender/sex	− .47***	− .34***	− .21***	− .56***	− .41***
Attractiveness^a^	.15***	.17***	.16***	.11***	.06***
Sobel test	− 3.34***	− 3.38***	− 3.37***	− 3.21**	− 2.69**
Mediation (%)	3	5	8	2	2

SDI-2 = Sexual Desire Inventory-2 (Spector et al., 1996); dyadic-P = dyadic (partner); dyadic-A = dyadic (attractive person). Simple and multiple regressions are each based on *N* = 5055 observations from participants that rated their attractiveness. Gender/sex was factor coded (0 = male, 1 = female). All continuous variables were z-standardized. ^a^Self-rated. ***p* < .01, ****p* < .001

**Table 16:** Coefficients of the mediation analyses: gender/sex–self-rated masculinity–sexual desire and Sobel Tests (Z-values)

Step 1: Regression of gender/sex on self-rated masculinity: − .67***					
Step 2–4: Regressions on sexual desire					
Predictor	SDI-2 score				
	Total	Dyadic	Dyadic-P	Dyadic-A	Solitary
Step 2: Gender/sex	− .48***	− .36***	− .23***	− .59***	− .41***
Step 3–4: Gender/sex	− .43***	− .32***	− .19***	− .53***	− .37***
Masculinity^a^	.07***	.06***	.05***	.06***	.06***
Sobel test	− 5.06***	− 4.17***	− 3.49***	− 4.34***	− 4.24***
Mediation (%)	10	11	15	7	10

SDI-2 = Sexual Desire Inventory-2 (Spector et al., 1996); dyadic-P = dyadic (partner); dyadic-A = dyadic (attractive person). Simple and multiple regressions are each based on *N* = 5035 observations from participants that rated their masculinity. Gender/sex was factor coded (0 = male, 1 = female). All continuous variables were z-standardized. ^a^Self-rated. ****p* < .001

**Table 17:** Simple regressions predicting total SDI-2 scores in a subsample of women prior menopause

Predictor	SDI-2 score				
	Total	Dyadic	Dyadic-P	Dyadic-A	Solitary
Hormonal contraception	−.04	.02	.08	−.13	−.16
P-value	.483	.669	.187	.035	.009

SDI-2 = Sexual Desire Inventory-2 (Spector et al., 1996); dyadic-P = dyadic (partner); dyadic-A = dyadic (attractive person). Simple regressions are each based on *N* = 1200 (*n* = 809 women without and *n* = 391 women with hormonal contraception) observations from female participants that indicated their use of hormonal contraception. Hormonal contraception was factor coded (0 = no use, 1 = use). All continuous variables were z-standardized

## Appendix D

See Figs. 4 and 5.Tables 18 and 19.

**Table 18:** Simple regressions predicting total SDI-2 scores

Predictor	*N*	Effect on total desire	Adj. R^2^
Gender/sex	8150	− .50***	.058
Age	8150	.11***	.011
Self-rated attractiveness	5054	.16***	.024
Self-rated masculinity	5036	.13***	.018
Self-rated health	4407	.05**	.002
Relationship status	4711	.12***	.003
Sexual orientation	7482	.20***	.007

SDI-2 = Sexual Desire Inventory-2 (Spector et al., 1996). Participant gender/sex, relationship status, and sexual orientation were factor coded (0 = male, 1 = female; 0 = single, 1 = in a committed relationship; 0 = heterosexual, 1 = same-sex attracted). All continuous variables were z-standardized. ***p* < .01, ****p* < .001

**Table 19:** Linear age trends predicting the SDI-2 scores

	Men		Women	
Outcome: SDI-2 score	Age Trend	Adj. R^2^	Age Trend	Adj. R^2^
Total	.12***	.015	.03	.001
Dyadic (Partner)	.09***	.008	.00	− .000
Dyadic (Attractive Person)	.08***	.006	− .03*	.001
Solitary	.10***	.010	.06***	.003

SDI-2 = Sexual Desire Inventory-2 (Spector et al., 1996). All continuous variables were z-standardized

^*^*p* < .05, ***p* < .01, ****p* < .001

## Appendix E

See Tables 20.

**Table 20:** Polynomial trends of sexual desire moderated by gender/sex

Predictor	Outcome: SDI-2 score				
	Total	Dyadic	Dyadic-P	Dyadic-A	Solitary
Gender/sex	− 0.45***	− 0.35***	− 0.21***	− 0.57***	− 0.38***
Age	10.16***	8.53***	7.92***	6.76***	8.06***
Age^2^	− 7.32***	− 5.87***	− 5.24***	− 5.22***	− 6.11***
Age^3^	2.08	2.79	3.45*	− 0.04	0.55***
Gender/sex × Age	− 9.28***	− 10.36***	− 8.75***	− 10.51***	− 3.71
Gender/sex × Age^2^	− 0.67	− 0.16	− 0.70	1.33	− 1.82
Gender/sex × Age^3^	1.29	− 1.83	− 2.91	1.73	4.79*
Adj. *R*^2^	.066	.038	.019	.082	.051

SDI-2 = Sexual Desire Inventory-2 (Spector et al., 1996); dyadic-P = dyadic (partner); dyadic-A = dyadic (attractive person). Polynomial regression models are each based on the observation of *N* = 8150 participants. Gender/sex was factor coded (0 = male, 1 = female). All continuous variables were z-standardized

**p* < .05, ****p* < .001

## Footnotes

1 To streamline our article, we do not report hypotheses on health and self-rated attractiveness here. However, full details of our hypotheses and corresponding results are given in our preregistration and Appendices, respectively.

2 Data collection continued for several years to maximize the sample size. Accordingly, the study link remained open as long as new individuals were interested in participating.

3 As can be seen from the analysis script (https://osf.io/q7n9x), we took 10,000 random resamples of our data, calculated Cohen’s *d* for the gender/sex comparison on each desire scale and then calculated the difference between the effect sizes. Finally, we calculated the confidence interval for this difference. If zero was not included in the confidence interval, we concluded that the effect sizes were significantly different from each other.
